# From Puffins to Plankton: A DNA-Based Analysis of a Seabird Food Chain in the Northern Gulf of Maine

**DOI:** 10.1371/journal.pone.0083152

**Published:** 2013-12-16

**Authors:** A. Kirsten Bowser, Antony W. Diamond, Jason A. Addison

**Affiliations:** 1 Department of Biology, University of New Brunswick, Fredericton, New Brunswick, Canada; 2 Atlantic Laboratory for Avian Research, University of New Brunswick, Fredericton, New Brunswick, Canada; Macquarie University, Australia

## Abstract

The predator-prey interactions within food chains are used to both characterize and understand ecosystems. Conventional methods of constructing food chains from visual identification of prey in predator diet can suffer from poor taxonomic resolution, misidentification, and bias against small or completely digestible prey. Next-generation sequencing (NGS) technology has become a powerful tool for diet reconstruction through barcoding of DNA in stomach content or fecal samples. Here we use multi-locus (16S and CO1) next-generation sequencing of DNA barcodes on the feces of Atlantic puffin (*Fratercula arctica*) chicks (n=65) and adults (n=64) and the stomach contents of their main prey, Atlantic herring (*Clupea harengus*, n=44) to investigate a previously studied food chain. We compared conventional and molecular-derived chick diet, tested the similarity between the diets of puffin adults and chicks, and determined whether herring prey can be detected in puffin diet samples. There was high variability in the coverage of prey groups between 16S and CO1 markers. We identified more unique prey with our 16S compared to CO1 barcoding markers (51 and 39 taxa respectively) with only 12 taxa identified by both genes. We found no significant difference between the 16S-identified diets of puffin adults (n=17) and chicks (n=41). Our molecular method is more taxonomically resolved and detected chick prey at higher frequencies than conventional field observations. Many likely planktonic prey of herring were detected in feces from puffin adults and chicks, highlighting the impact secondary consumption may have on the interpretation of molecular dietary analysis. This study represents the first simultaneous molecular investigation into the diet of multiple components of a food chain and highlights the utility of a multi-locus approach to diet reconstruction that is broadly applicable to food web analysis.

## Introduction

Ecosystems are characterized and understood from the fundamental relationships between predator and prey. When linked vertically and horizontally these relationships form food webs, which depict how energy flows through ecosystems and demonstrate how various components of the web interact. The consideration of an ecosystem in its entirety, and not simply an assemblage of independent parts, is the cornerstone of the widely-accepted ecosystem-based management paradigm [1,2,3,4,5,6,7], which relies on accurate assessments of food webs. Since our understanding of ecosystem functioning depends on the data within a food chain, outdated, biased, or incomplete assessments of diet weaken our ability to predict how and why changes to ecosystems occur. 

Conventional methods of constructing food chains rely on diet studies which visually identify prey during feeding events or within stomachs, pellets, or feces. These techniques often suffer from misidentification of similar-looking prey, underrepresentation of soft-bodied (within stomach contents) or small (when observed in the field) prey, and low taxonomic resolution, where prey cannot be precisely identified due to distance (during observations) or digestion (within stomach contents) [8]. Biochemical methods such as fatty acids, stable isotopes, and DNA have gained popularity in diet studies and can be used as a form of quality control for conventional techniques. However, these methods offer insight on diet in the longer term and are not necessarily well suited for prey species identification, particularly when there is little *a priori* knowledge of diet. The use of DNA within feces or stomach contents provides a snapshot of predator diet and has not only been shown to provide a better estimate of diet than conventional methods [9,10,11,12], but can also provide species-level prey identification through the use of publicly available reference sequences. DNA-based techniques are advantageous in that they can be used either for comparative purposes for previously known diets or for *de novo* diet description.

The application of DNA barcoding in diet studies has increased considerably with the advent of next-generation sequencing (NGS) technology. It is now possible to identify even the rarest prey from multiple predators to species, genus, or family level in a single sequencing run while maintaining the ability to trace back each prey to the sample from which it came. NGS has been used in barcoding diet studies for fur seals [13], little penguins [14,15], slow worms [16], bats [17,18], leopard cats [19] and tapirs [20]. However prey identification from DNA in diet samples can be greatly influenced by technical issues including the uncertainty about the taxonomic diversity expected in the sample and the poor quality of the genomic DNA, particularly when extracted from fecal samples. Most DNA-based diet studies design multiple group-specific primers that amplify the various prey types in predator diet, but these studies may fail to describe the full taxonomic range of the prey consumed. Universal primers can amplify and resolve species across a broad variety of taxa, making them a good, cost- and time-effective alternative to group-specific primers. Using multiple markers may also provide a broader taxonomic resolution of diet as different markers are not suitable barcodes for all taxonomic groups (e.g. 21). In addition, PCR amplification of degraded DNA is more reliable when target fragments are small [22]. Moreover, up to 90% of the sequences obtained from NGS [19] can be less-degraded host DNA. The inclusion of primers to block host DNA amplification can increase the number of prey sequences significantly. Finally, it is also possible that the results may be misleading if primers can amplify the prey within the prey on which predators feed (i.e., secondary consumption). This may skew the interpretation of how those species interact with the rest of the ecosystem. Thus a comparison of the diet of both predator and prey is warranted to assess the potential for detection of secondary consumption by DNA based methods. To date, no diet studies employing NGS have investigated multiple components of a food chain. 

Here we use Atlantic puffins (*Fratercula arctica*) of Machias Seal Island and their main prey, Atlantic herring (*Clupea harengus*) as a model system to investigate how molecular methods can be used to describe food chains as these two species represent a simple food web where two conventional methods of studying diet have been employed. Puffin chick diet is known from hundreds of hours of observing adults provisioning their chicks as part of a long-term seabird research program [23] which will provide a comparison for molecularly-derived chick diet. By investigating the diet of the major prey of puffins (herring), we can also consider the potential effect of secondary consumption. It is also possible for molecular methods to shed light on questions that have historically been challenging or impossible to answer. For instance, as adult puffins forage at sea and do not leave identifiable components of prey in feces or in the form of a pellet, chick diet has been used as a best estimate for adult diet for birds in the Gulf of Maine, an assumption supported by similar levels of nitrogen isotopes in chick and adult blood (same trophic position) [24]. However, theory on optimal foraging predicts that as central-place foragers, adult puffins should feed their chicks a less diverse diet of high quality food while they feed on a more varied assortment of potentially lower quality prey [25]. DNA-based dietary analysis of fecal samples offers the opportunity to document Gulf of Maine adult puffin diet and to test the similarity between adult and chick diet. Additionally, the diet of the main prey of puffin chicks (juvenile herring) is not well known and based on five >30 year old stomach content analyses [26,27,28,29,30]. Molecular-derived herring diet can simultaneously evaluate conventional stomach content analyses of diet while extending our knowledge of an important ecosystem.

In this paper we apply next-generation sequencing of DNA barcodes from two genetic markers on puffin adult and chick fecal samples and herring stomach contents to study diet and describe the food chain in which these species exist. We compare the diet of puffin chicks using conventional field observations and molecular methods, test the assumption of common diet in puffin adults and chicks, and test for the effect of secondary consumption by considering the diets of chicks and herring. Further, we use the prey species detected by each genetic marker in diet samples to assess the efficacy of a multi-locus technique as well as to make inferences about foraging ecology of puffins and herring. Since food webs provide the framework from which we draw conclusions about how an ecosystem functions, it is imperative that the studies used to construct food webs provide the most accurate, unbiased, and repeatable estimated of diet possible. This paper provides the first analysis of multiple genetic markers across multiple taxa within a marine food web and demonstrates the broad utility of the technique as a tool for diet reconstruction in fish and birds.

## Methods

### Ethics Statement

This study was approved by the University of New Brunswick’s Animal Care Committee (Animal Care Permit No. 09012) as well as the Canadian Wildlife Service (Scientific Take Permit No. ST2642 for handling puffins and MBS/MSI 09-6 for disturbing birds in a protected, federally-owned Migratory Bird Sanctuary).

### Sampling

To assess the effectiveness of conventional field observations as an appropriate method to study puffin diet, we compared diet as estimated from 68 hours of field observations and through DNA within fecal samples of puffin chicks in the 2009 breeding season (May-August) on Machias Seal Island (44°30’N, 67°6’W), New Brunswick, Canada. Field observations were conducted in three hour stints with binoculars from observation blinds according to Machias Seal Island’s research protocols [23,31] where the number, length, and species or category (e.g. “krill”) of all prey were recorded in each bill load delivery of a provisioning adult. A total of 91 chick fecal samples were collected opportunistically during regular research activities. To test the similarity between the diets of puffin adults and chicks we collected 10 adult fecal samples per week (total 146) throughout the season. Fecal samples were stored in 70% ethanol. To assess herring diet and to investigate the potential effect of secondary consumption, we dissected the stomachs from 77 collected juvenile herring (~5-15cm) that been dropped accidentally by provisioning seabird adults. Other prey found within the colony, as well as several invertebrate and fish species obtained from the island’s intertidal zone and from refuse buckets donated by passing fishermen, were stored in Whirl-pak® bags (Nasco) at -20°C to generate a sequence database of local fauna from which fecal and stomach DNA could be identified. All fecal samples and stomach contents were stored in 5-10ml 70% ethanol at 4°C for three months, -20°C for four months, and then -80°C for two years. 

### Primer Design

Short target amplicons are preferred for PCR-based molecular diet analysis because fecal DNA is often highly degraded [22,32]. We used universal primer pairs to target small (~130-300bp) fragments of the mitochondrial genes 16S and cytochrome *c* oxidase subunit 1 (CO1). The degenerate primers 16S1F and 16S2R amplify a ~180-270bp region of 16S and have been shown to amplify prey DNA from the feces of penguins (e.g., fish, euphausiids, squid [12]). To complement these data, we employed a second set of universal primers [33] that successfully amplifies a 130bp region of the CO1 in over 600 species of mammals, fishes, birds, and insects, making it a good candidate to detect species not amplified by 16S (Table 1). 

**Table 1:** Universal primers for 16S and CO1 genes with associated blocking primers.

**Primers**	**Sequence 5'-3'**	**Reference**
16S1F	GACGAKAAGACCCTA	[12]
Herring Blocker	*GACCCTA*TGGAGCTTTAGACGCCCAC3	This study
16S2R	CGCTGTTATCCCTADRGTAACT	[12]
Puffin Blocker	*CCCTGGGGTAGCT*TGGTCCATTGATCC3	This study
Uni-MinibarF1	TCCACTAATCACAARGATATTGGTAC	[33]
Uni-MinibarR1	GAAAATCATAATGAAGGCATGAGC	[33]

Italics on 5’ region of blocking primers depict area of overlap on 3’ end of universal primer.

We used a pooled massively parallel sequencing (MPS) approach following the protocol of Puritz et al. [34]. To allow recovery of sample identification from sequence data we included a 10bp multiplex identifier (MID) tag between the Lib-L 454 sequencing adapter (26bp plus a 4bp signal calibration key) and the universal primer (16S or CO1) in our custom engineered forward and reverse primers (Table 2). 

**Table 2:** Next generation (454) sequencing adapters and MID tags used in sequencing primer design.

**Lib-L 454 Sequencing Adapters and Key (5'-3')**	
Forward	CCATCTCATCCCTGCGTGTCTCCGACtcag
Reverse	CCTATCCCCTGTGTGCCTTGGCAGTCtcag
**Multiplex Identifier Tags (5'-3')**	
MID-1	ACGAGTGCGT
MID-2	ACGCTCGACA
MID-3	AGACGCACTC
MID-4	AGCACTGTAG
MID-5	ATCAGACACG
MID-6	ATATCGCGAG
MID-7	CGTGTCTCTA
MID-8	CTCGCGTGTC
MID-9	TAGTATCAGC
MID-10	TCTCTATGCG
MID-11	TGATACGTCT
MID-12	TACTGAGCTA
MID-13	CATAGTAGTG
MID-14	CGAGAGATAC
MID-15	ATACGACGTA
MID-16	TCACGTACTA

Stomach and fecal samples can contain significant amounts of DNA from the host species due to sloughing of cells in the digestive tract [19]. Host DNA may represent a huge proportion of the total number of sequences obtained through sequencing, reducing the detection of prey with little or difficult-to-amplify DNA [19]. Preliminary sequencing of cloned16S PCR products indicated a high frequency of host DNA sequences recovered from both stomach and fecal samples. Thus, we designed herring and puffin blocking primers for 16S1F and 16S2R (respectively) using the C3 spacer method described by Vestheim and Jarman [35] (Table 1). Blocking primer efficiency was tested with puffin and herring DNA in isolation, and under competitive conditions in PCR reactions consisting of mixtures of prey (our reference samples) and predator (puffin or herring) DNA. Although we did not identify interference between the blocking primers and any of our reference samples, it is possible that the blocking primer (overlapping a highly conserved region of DNA) prevented ideal amplification of prey types that we were unable to asses. Blocking primers were used with 16S amplifications at a blocking to universal primer ratio of 5:1. The use of a blocking primer to enrich the genomic DNA samples for rare prey templates reduced the proportion of herring sequences by 86% and doubled the number of taxa identified (from 10 to 20 taxa) in a test of eight 16S-amplified herring stomach contents (data not shown).

### DNA Extraction and Amplification

DNA from stomach contents and reference samples was isolated with a CTAB protocol [36]. DNA was resuspended in 20-100 ul TE (Ambion pH 8.0) and concentration and purity were evaluated with NanovueTM (General Electric Life Sciences). Fecal samples were centrifuged for 30 minutes at 4°C and storage ethanol was poured off. DNA was extracted with QiaAmp DNA Stool Mini Kit (Qiagen). Samples with small amounts of fecal material were eluted with 75-100ul of buffer AE instead of the recommended 200ul. DNA was stored in 2ml microcentrifuge tubes at -20°C.

Amplification of fecal DNA with 16S MID-tagged sequencing primers was achieved in 30ul reactions containing 6ul undiluted template, 0.2mM dNTP, 1X bovine serum albumin (BSA; New England Biolabs), 5mM MgSO4, 0.25uM of each primer, and 1.25 of blocking primer, 1X High Fidelity Buffer, and 1.2 units Platinum® Taq DNA Polymerase High Fidelity (Life Technologies). DNA from fish stomach contents was amplified with 16S MID-tagged sequencing primers in 30ul reactions containing 3ul undiluted template, 0.2mM dNTP, 5mM MgSO4, 0.25 uM of each primer, 1.25uM of blocking primer, 1X High Fidelity Buffer, and 1.2 units Platinum® Taq DNA Polymerase High Fidelity. DNA from samples intended for the reference database of potential prey was amplified with 10ng of template, 0.2mM dNTP, 0.5mM MgSO4, 0.25uM of each forward and reverse 16S primer, 0.04 units of Taq DNA polymerase, and 1X ThermoPol Buffer (New England Biolabs). Thermocycling protocol for 16S began at 94°C for 10 minutes followed by 35 cycles of 94°C for 15s, 55°C for 15s, and 68°C for 30s, with a final extension of 68°C for 5 minutes (BioRad C-1000). 

Amplification with CO1 for both sample types followed similar component and cycling conditions as the 16S with the following modifications: 3ul undiluted template was used, BSA was omitted, there was no blocking primer, and annealing temperature was set at 53°C. All amplicons were visualized under UV light in 2% agarose using SYBR Safe (Life Technologies), and those that amplified were cleaned with Agencourt AMPure XP (Beckman Coulter) at a 0.9 beads to 1 PCR product ratio. DNA concentration was determined with dsDNA BR assays on a Qubit 2.0 Fluorometer (Life Technologies). PCR was repeated for samples with less than 20ng of 16S-amplified DNA then pooled after cleaning with QIAEX II Gel Extraction Kit following the manufacturer’s instructions.

All pipetting was done with barrier tips, negative controls were used in every PCR, small aliquots of reagents were used, and lab benches were cleaned between uses. Adult, chick, and herring diet samples were DNA-extracted on different days, several weeks in advance of the PCR component of this project.

We made considerable efforts to obtain a sufficient number and an even temporal distribution of samples throughout the sampling period. However, a limiting step in our protocol was the extraction of high quality DNA. Amplification success of herring stomach contents and puffin fecal samples ranged from 47% to 71%. Insufficient homogenization during DNA extraction and PCR inhibitors are two potential explanations for the large number of samples that could not be successfully PCR amplified. There was also considerable variation in sample coverage. Although sequencing success of submitted samples was over 94%, only 61-84% of these samples produced a sufficient number of sequences to be included in calculations of frequency of occurrence (see next section). This variation may be a result of MID tag-induced amplification and sequencing bias [37], imprecision in equimolar pooling of amplicons, or variability in sample quality (as both fecal and stomach samples are likely to contain many PCR-inhibitors).

### Sequencing, Bioinformatics and Analysis

Samples were quantified and pooled into a single equimolar library and sequenced by Génome Québec Innovation Centre at McGill University in Montreal, Quebec, Canada. The library was sequenced unidirectionally on half a pico titre plate using the Roche GS-FLX (454) platform. Bases were recalled using Pyrobayes [38] and sequences were filtered for length (40-400bp) and quality (mean Q20 across fragment) using Prinseq [39]. Sequences were then demultiplexed based on their MID tag combinations (exact matches only) and divided by amplicon type (16S or CO1, exact matches only) with jMHC [40]. The program jMOTU [41] was used to assemble sequences into molecular operational taxonomic units (MOTUs) at various levels of base pair differences (cutoffs). Sequences were clustered into MOTUs with cutoff ranges from 1-30 base pairs (and suggested gathering parameters of 95% low BLAST identity filter and default settings for sequence alignment overlap). At increasing cutoff values (number of base pairs different between sequences), the number of MOTU produced by jMOTU decreases and eventually reaches an asymptote. Cutoffs were chosen at the beginning of this asymptote to ensure a high degree of taxonomic diversity at the expense of producing multiple MOTUs belonging to a single taxon. MOTUs at the chosen cutoff were exported to an sql file, which was uploaded to the relational database management software, PostgreSQL (hwww.postgresql.org). Representative sequences were chosen as the first sequence appearing in jMHC data output belonging to a unique MOTU. All CO1 representative MOTUs were queried using Barcode of Life Database (BOLD) online identification tool [42]. All representative MOTUs that were not identified to species by BOLD’s online tool were assigned to a taxanomic level that incorporated all potential matches to the queried sequence at 100% identity. MEGAN [43] was used to corroborate BOLD-based taxon assignments as well as to assign taxonomy to representative MOTUs with no match to BOLD. BLAST files (BLASTn, queried 22 March, 2013 with default algorithm parameters) were imported to MEGAN (min score=75, min support=2, top percent=10, min complexity=0) and the MEGAN taxon assignments were accepted or rejected based on the % identities of each MOTU’s top matches. MOTUs were assigned to the top match if it was 96% similar or higher and was a possible component of this study system, otherwise MOTUs were assigned to the most highly resolved taxonomic node common to all significant matches. Similar criteria were used for taxon assignment for 16S MOTUs except we also included the sequences generated from our reference samples in our consideration of taxon assignment. 

We used PRIMER 6 (PRIMER-E Ltd) [44] for multivariate statistical analyses. We used an Analysis of Similarity (ANOSIM) to compare chick diet with method type (field observation or DNA) as a fixed factor. We also conducted an ANOSIM to test the difference between puffin diet with age (chick or adult) as a fixed factor. Data were converted to binary (presence/absence for fecal samples or for field observation provisioning deliveries). Resemblance matrices were built from Bray-Curtis similarity, from which the ANOSIM was run with 999 permutations. We used non-metric multidimensional scaling (MDS, 100 restarts) with overlaid vectors representing the correlations (Pearson correlation coefficients, >0.35 for chick/adult comparisons, all for diet by method (DNA/field)) to visualize differences between diets or chick and adults and between diet studying methods. All MDS graphs had stress values below 0.2 [45].

We used the frequency with which a prey was detected across samples, the frequency of occurrence (FOO), to quantify diet because the proportion of prey sequences found within a sample does not necessarily correlate quantitatively with proportions of prey consumed [14]. We limited FOO calculations to samples containing at least 50 sequences per marker for each of the three sample types obtained within the common sampling period (the time frame where all sample types were available for collection, 13 June to 29 July). When a taxon was identified with both 16S and CO1, we used all samples (that met above criteria) to estimate FOO, otherwise FOO was calculated based on number of samples amplified by the respective gene. Table S4 outlines gene-specific sample size and sequencing success as well as which data are used in the creation of various figures and tables.

## Results

After initial filtering for quality and length, 104,313(16S) and 49,218 (CO1) reads were demultiplexed with over 98% (16S) and 96% (CO1) of sequences successfully matched to herring stomach content samples (n=44) or fecal samples from puffin adults (n=64) and chicks (n=65). We assembled 5759 unique CO1 amplicons >80bp into 132 MOTUs at the 11bp cutoff and 16, 710 unique 16S amplicons >75bp were assembled into 273 MOTUs at the 12bp cutoff. MOTUs were identified to 78 unique prey taxa from kingdom Animalia, and one green alga, one brown alga, three diatoms, two water molds, three bacteria, a protozoan, and several contaminating taxa (gull, human, etc.). All non-contaminant identified taxa are listed in Table S1. Sequences that could not be assigned to a taxonomic kingdom or had no hits when queried with BLAST were omitted (25% and 20% of 16S and CO1 MOTU). 

**Figure 1:**
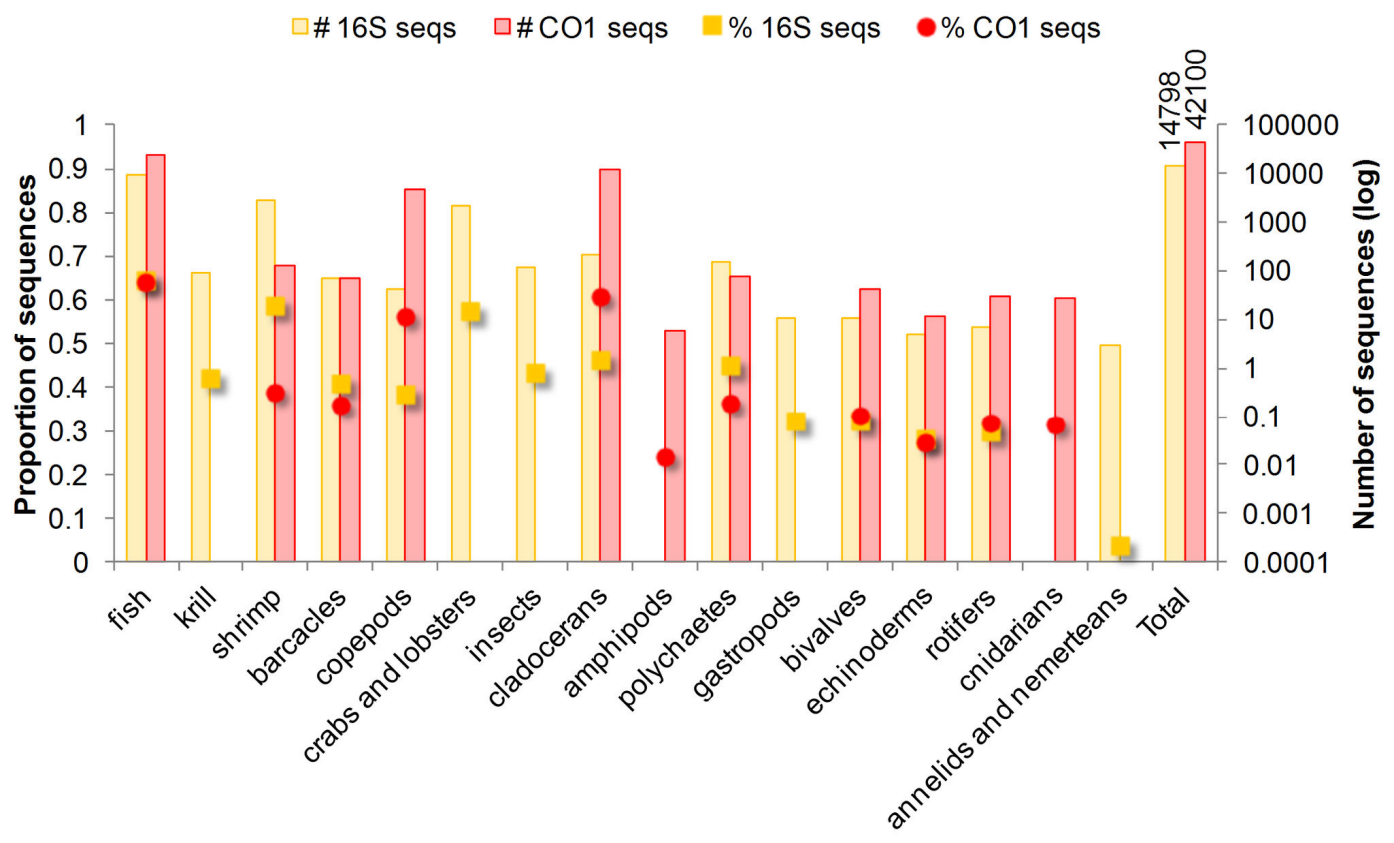
Coverage comparison of 16S and CO1 barcoding markers on prey groups. Number (right, log scale) and proportion (left) of sequences belonging to various types of identified prey taxa from 16 puffin adult, 22 puffin chick, and 28 herring samples. All samples produced at least one sequence for each gene. Only 20 (8 adult, 9 chick, 3 herring) of the 66 samples produced over 50 sequences per marker (Table 3).

**Figure 2:**
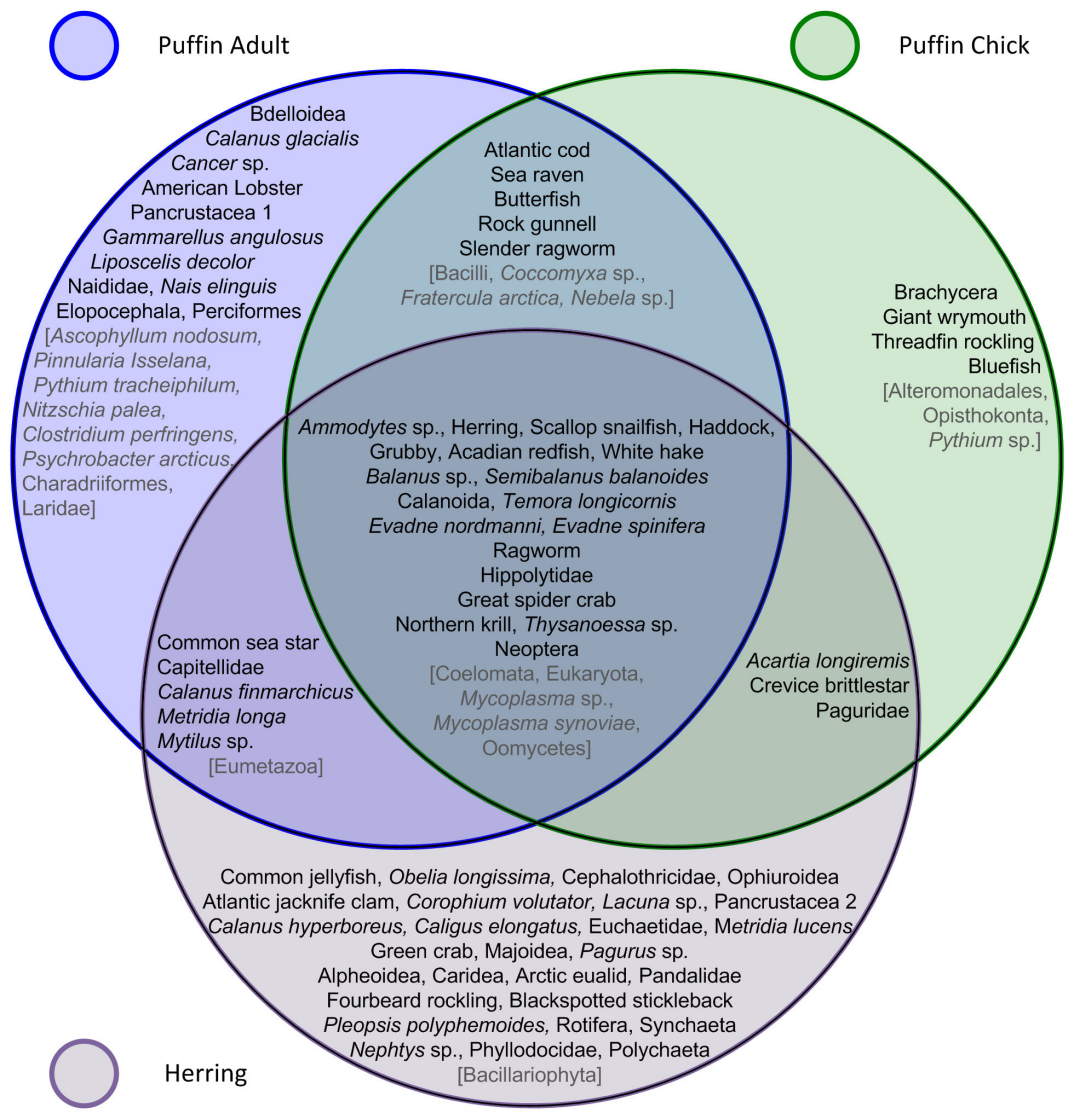
Summary of taxa identified from all sequenced puffin adult and chick fecal samples and from all sequenced herring stomach contents. Overlapping regions represent taxa occurring in multiple sample types (eg: centre region contains taxa found in all three predator diet samples). Non-animal and non-informative taxa are listed in grey text in square brackets.

We identified more unique prey with 16S than CO1 barcoding markers (51 and 39 taxa, Table S1). Only 12 taxa were identified by both markers but often there were large discrepancies in both the coverage and FOO (Table 3). Herring had the largest number of sequencing reads within the twenty samples that produced at least 50 sequences from each marker, followed by Acadian redfish (*Sebastes fasciatus*). An additional 46 samples contained at least one amplicon from each locus. Within these 66 samples, coverage of krill, shrimp, crabs and lobsters, insects, gastropods, and annelids and nemerteans was higher with 16S (Figure 1). Conversely, CO1 had higher coverage of fish, copepods, cladocerans, amphipods, rotifers, and cnidarians.All taxa found in diet samples from our three predators (regardless of coverage or time period) are displayed in Figure 2. Herring stomach samples had the most diverse assemblage of taxa. Many of the taxa in herring diet are suspected to be the planktonic stages of marine invertebrates, with the exception of a polychaete without a planktonic larval phase [46] (Ragworm, *Hediste diversicolor*) and possibly a snail from the *Lacuna* genus which has one known Gulf of Maine representative with direct development [47]. Copepods and decapods are the dominant prey types; however herring also appear to be predators of echinoderms, cnidarians, bivalves, gastropods and at least eight fish species.

**Table 3:** Comparison of sequence amplification and frequency of occurrence (FOO) of common taxa in puffin adult, puffin chick, and herring samples amplified with both markers.

		**Adult (n=8)**		**Chick (n=9)**		**Herring (n=3)**	
**Common Taxon**		**16S**	**CO1**	**16S**	**CO1**	**16S**	**CO1**
*Ammodytes* sp.	FOO (%)	37.5	25	44.4	22.2	-	-
	# Seqs	60	12	172	32	0	0
*Balanus* sp.	FOO (%)	12.5	0	-	-	66.7	33.3
	# Seqs	2	0	0	0	22	33
Calanoida	FOO (%)	-	-	0	22.2	0	33.3
	# Seqs	0	0	0	66	0	1
Caridea	FOO (%)	-	-	-	-	33.3	33.3
	# Seqs	0	0	0	0	4	2
Atlantic herring	FOO (%)	100	100	100	100	100	100
	# Seqs	4869	6880	8980	3826	329	11579
*Eualus fabricii*	FOO (%)	-	-	-	-	33.3	33.3
	# Seqs	0	0	0	0	1	27
*Evadne nordmanni*	FOO (%)	0	37.5	0	44.4	66.7	66.7
	# Seqs	0	6	0	34	290	197
Atlantic puffin	FOO (%)	25	0	22.2	22.2	0	0
	# Seqs	12	0	2	4	0	0
Pancrustacea	FOO (%)	-	-	-	-	0	33.3
	# Seqs	0	0	0	0	0	5
Acadian redfish	FOO (%)	0	62.5	0	33.3	0	33.3
	# Seqs	0	2810	0	5306	0	7
*Semibalanus balanoides*	FOO (%)	-	-	0	22.2	66.7	66.7
	# Seqs	0	0	0	6	64	11
White hake	FOO (%)	25	37.5	22.2	33.3	0	66.7
	# Seqs	2	18	2	16	0	10

Twelve common taxa were identified by both 16S and CO1 markers. Dashes are used when taxa are absent from a sample (not detected in these samples by either gene). Zeros represent the failure of one marker to detect a taxon. Adult (n=8), chick (n=9), and herring (n=3) samples contain at least 50 sequences per sample per marker.

Many of the invertebrate species found in adult and chick puffin fecal samples were also observed in herring (Figure 2). Four species of fish and a polychaete, the Slender ragworm (*Nereis pelagica*), were detected in both puffin sample types. Only adult puffin fecal samples contained representatives from the annelid class Clitellata and two unidentified fish taxa (Elopocephala, Perciformes) plus an assortment of invertebrate taxa. The assemblage of prey found exclusively in chick diet was less varied than that of adults, comprising three species of fish and a fly that the chicks presumably eat while in their nest burrow (Brachycera). 

**Figure 3:**
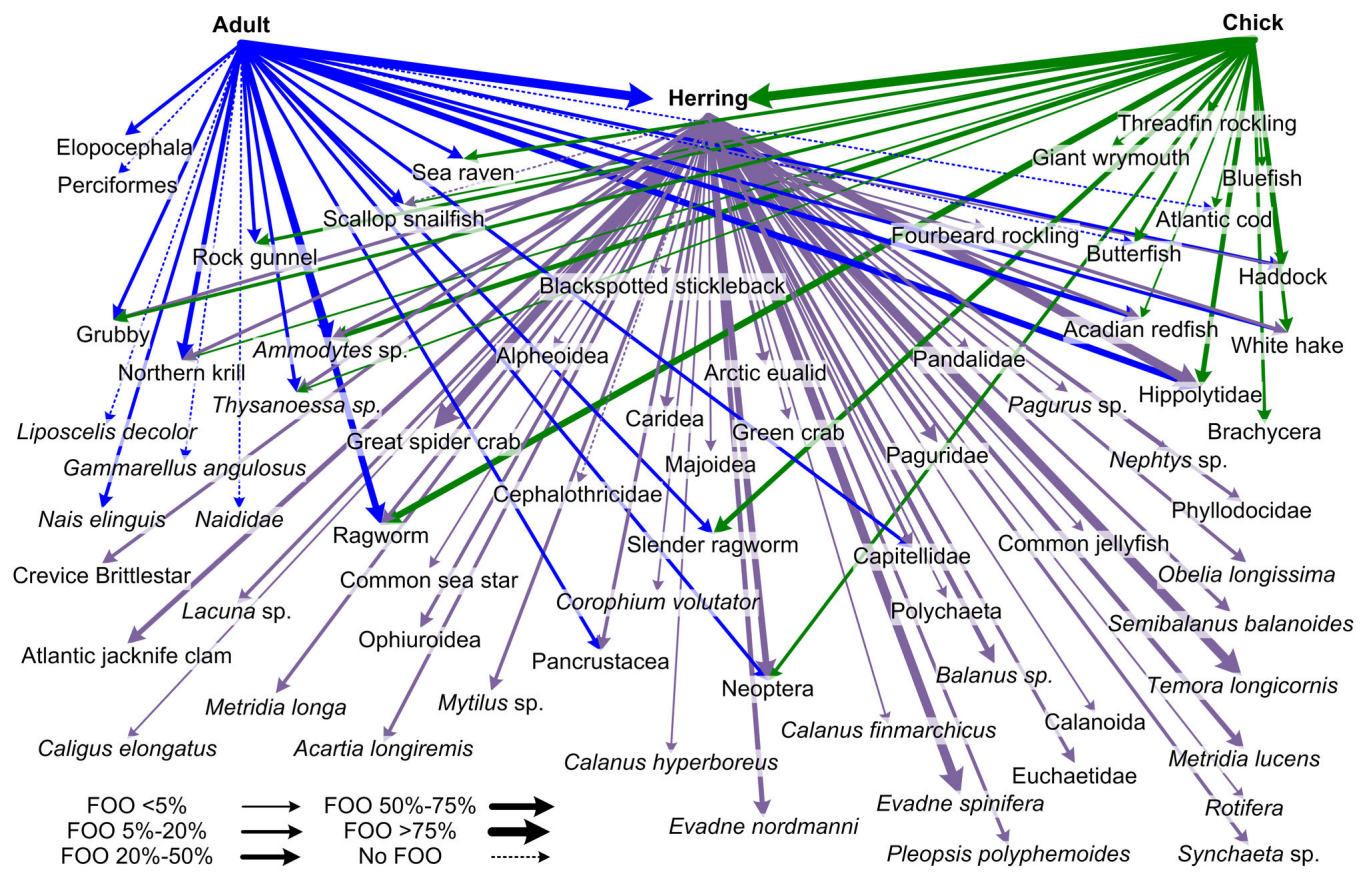
A graphical representation of the Machias Seal Island Atlantic puffin (*Fratercula arctica*) and Atlantic herring (*Clupea harengus*) food chain. Increasing line thickness relates to higher frequency of occurrence in adult (blue), chick (green), and herring (purple) diet. Data are all derived from samples within common sampling period (13 June - 29 July).

**Figure 4:**
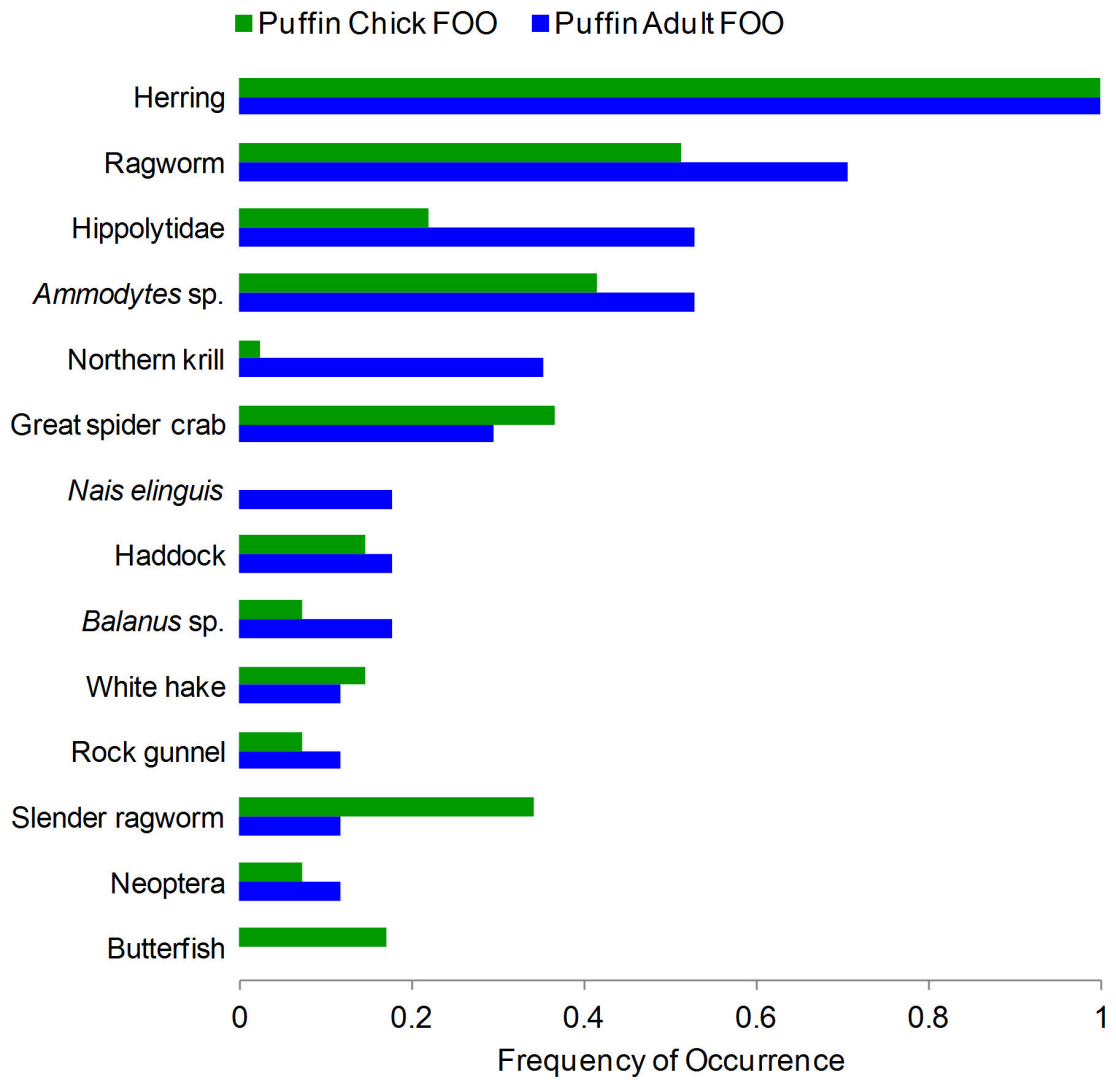
Frequency of occurrence (FOO) of top 16S taxa (>10%) in diet of puffin adults and chicks. Blue bars represent adult (n=17) data, green bars represent chick (n=41) data.

Quantitative description of puffin and herring diet was based on the frequency of occurrence of taxa in samples with at least 50 sequences obtained within the common sampling period (13 June to 29 July, Table S1). Taxa observed in over 20% of all herring samples include: shrimp (Hippolytidae 92%), cladocerans (*Evadne spinifera* 100% and *E. nordmanni* 43%), crabs (Great spider crab, *Hyas araneus* 84% and Paguridae 24%), copepods (*Temora longicornis* 87% and *Mertridia lucens* 28%), barnacles (*Balanus* sp. 46%), insects (Neoptera, 56%), the Atlantic jackknife clam (*Ensis directus* 22%), and Northern krill (*Meganyctiphanes norvegica* 20%) (Figure 3). Herring were present in all adult and chick puffin samples (Figure 4). The next most frequently observed fish was detected in just over one-third of puffin samples (*Ammodytes* sp. 33% for adults, 37% for chicks,). Acadian redfish were present in roughly one-third of all adult samples but only 4% of chick samples. Ragworms were detected more often in adult diet (adults71%, chicks 51%) whereas the Slender ragworm was found in only 12% of adult samples but in 34% of chick samples. Shrimp and krill were much more prevalent in adult than chick diet (Hippolytidae: adults 53%, chicks 22%; Northern krill: adult 35%, chick 2%). We also detected taxa (over 20% of all samples) that are unlikely or impossible target prey for adult and chick puffins (Great spider crab, the copepod *Temora longicornis*, and the cladoceran *E. spinifera*, see Figure 2). 

**Figure 5:**
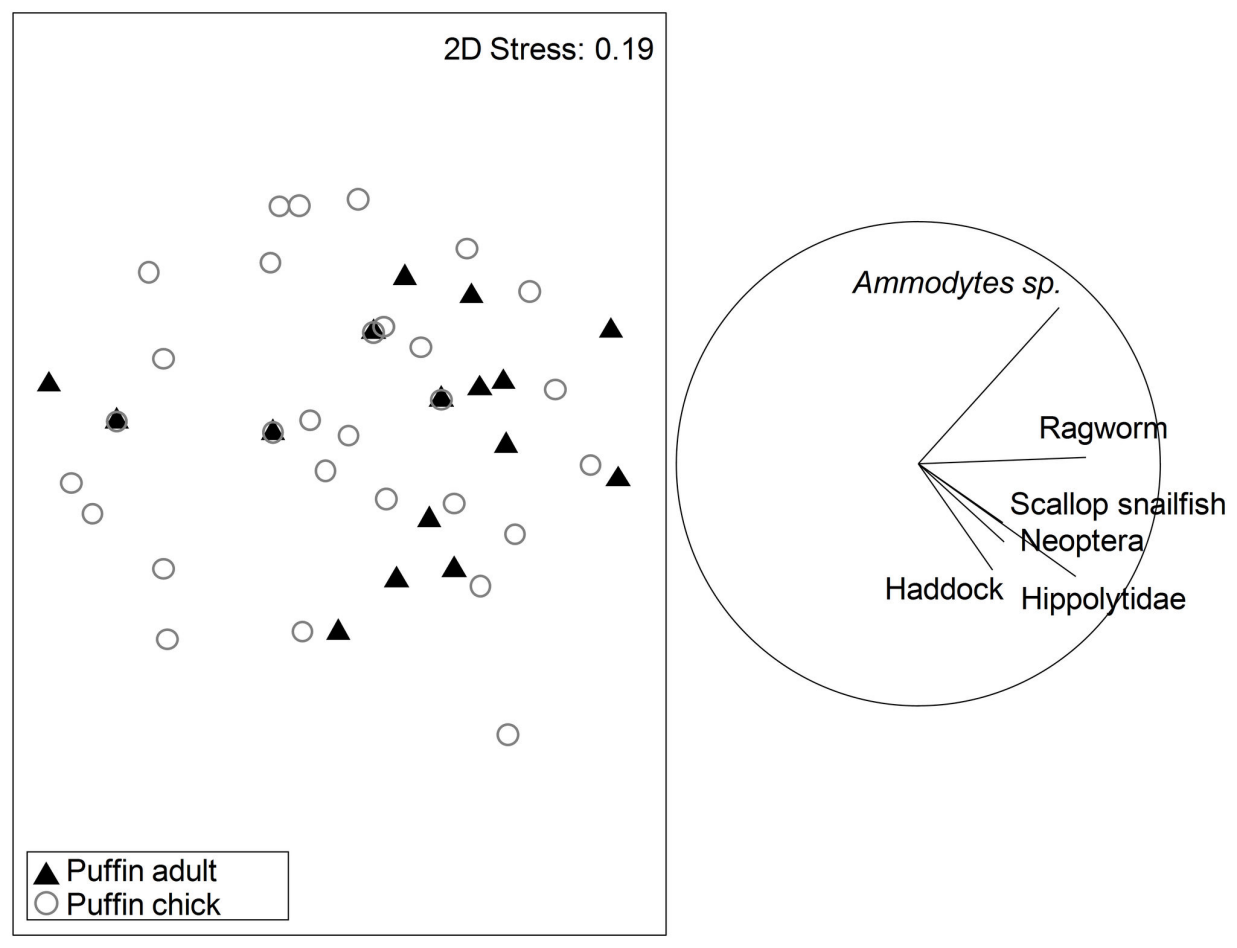
Non-metric multidimensional scaling (MDS) plots depicting differences in prey assemblage of adult and chick puffin diet. Analysis was limited to 16S samples with >50 sequences per fecal sample and taxa which could be considered targeted prey for puffins. Each symbol represents a fecal sample, and the distance between the different symbols represents the difference in taxa composition between fecal samples. There is much overlap between adult and chick fecal samples, reflecting diet overlap. Vectors are the correlations >0.35 between a prey taxon and the MDS axes, where vector length and direction reflect taxon frequency; the big circle indicates the maximum vector length.

The diets of puffin adults (n=17, 12 samples omitted due to lack of diet-informative taxa) and of puffin chicks (n=41) could not be distinguished statistically (ANOSIM, Global R = 0.079, p=0.058). The analysis was limited to only 16S-identified animal taxa (n=40) and puffin fecal samples collected within the common sampling period (13 June – 29 July) from which a minimum of 50 sequences were obtained (Figure 4). The similarity between the diets of adults and chicks was confirmed, when 14 taxa unlikely to have been targeted puffin prey (*Acartia longiremis*, *Balanus* sp., Calanoida, *Calanus finmarchicus*, *Calanus glacialis*, *Cancer* sp., *Evadne nordmanni*, *Homarus americanus*, Great spider crab, *Metridia longa*, *Mytilus* sp., *Ophiopholis aculeata*, Paguridae, *Semibalanus balanoides*), were removed (Global R = 0.07, p=0.085, Figure 5). 

**Figure 6:**
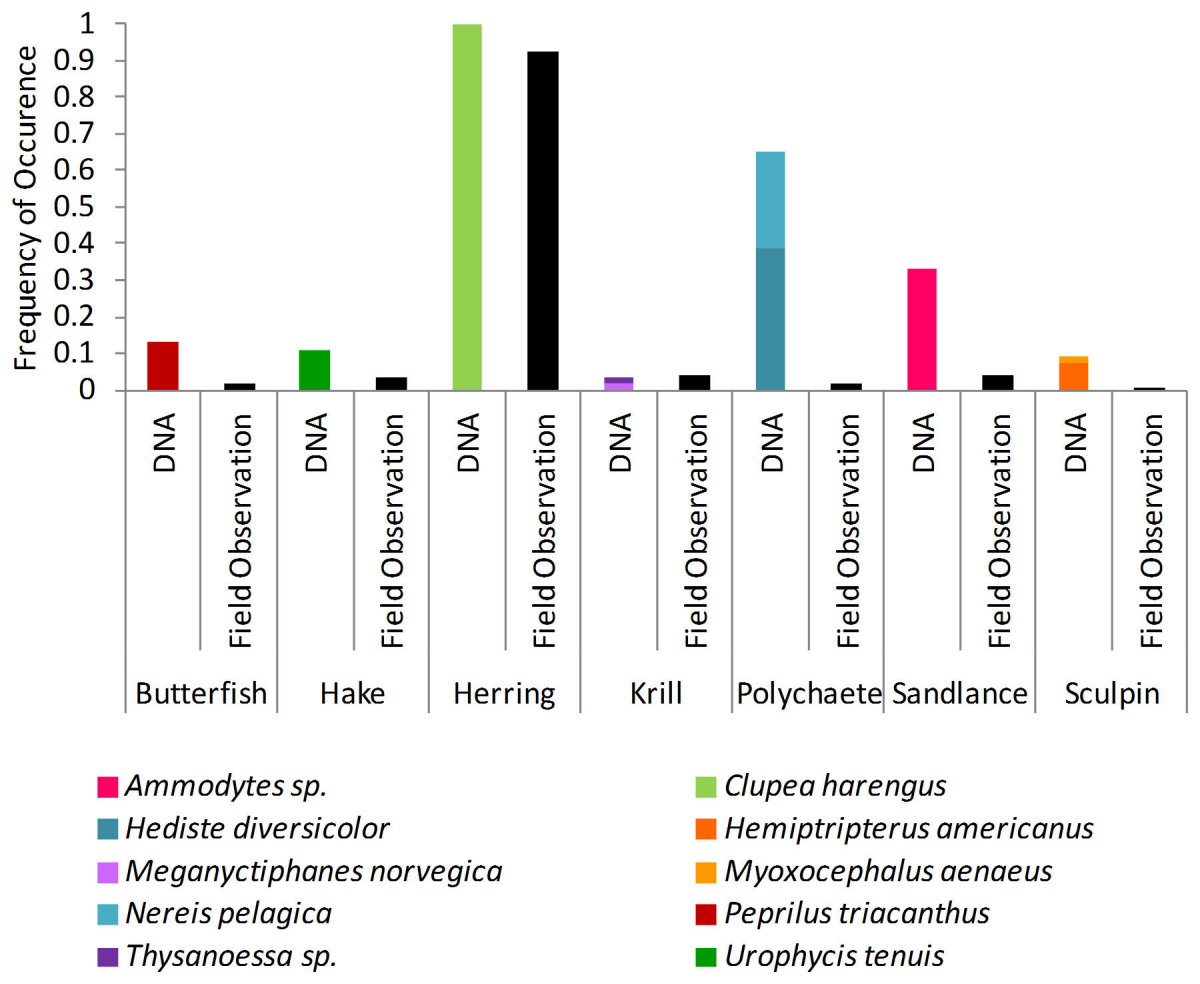
Frequency of occurrence of chick prey through field observations and DNA sequencing. Coloured bars represent DNA-identified taxa which are assigned to one of 7 field observation prey categories (black bars).

**Figure 7:**
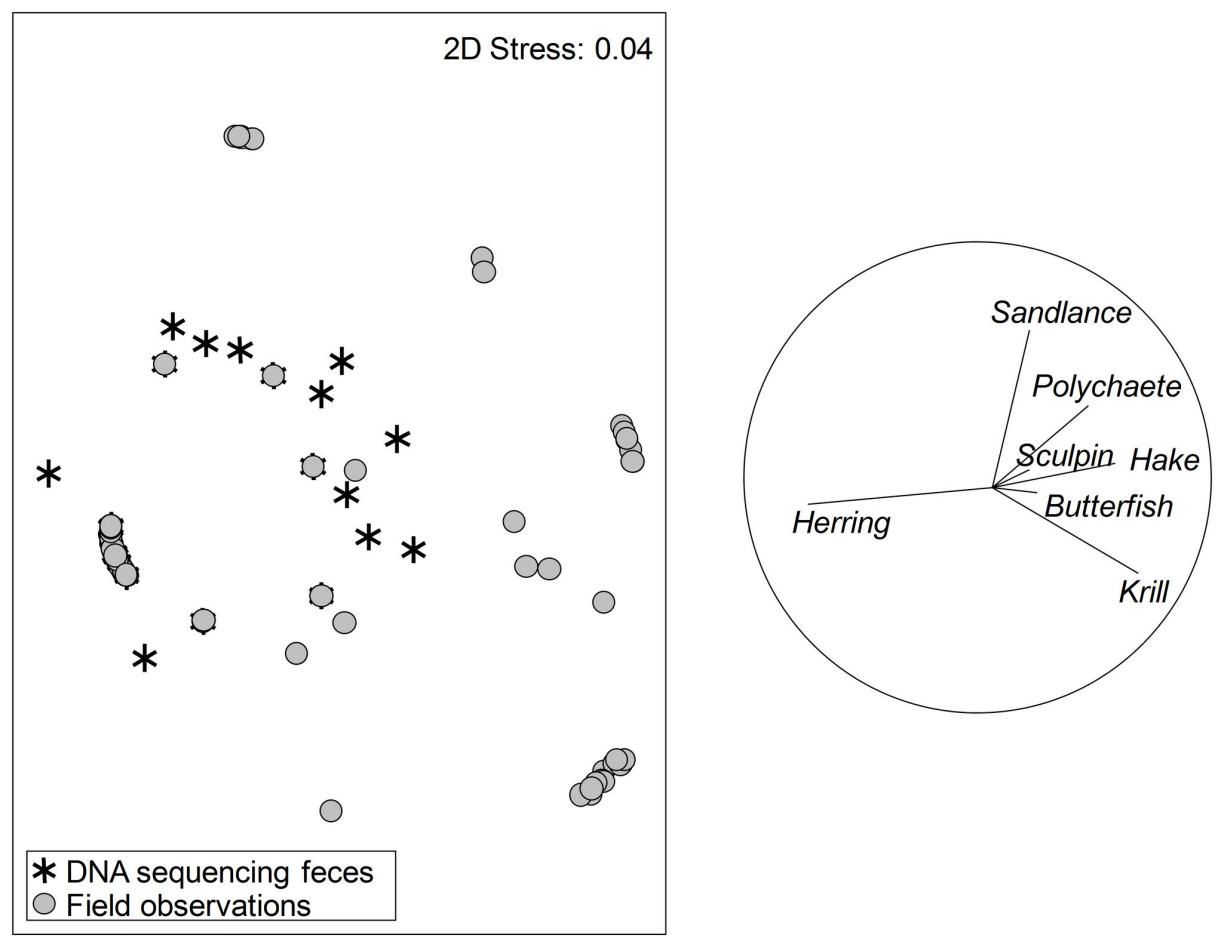
Non-metric multidimensional scaling (MDS) plots depicting differences in prey assemblage of chick diet through DNA and field observations. Grey circles represent a single feeding delivery to a puffin chick. Stars represent individual chick fecal samples. Vectors are the correlations between prey types (herring, sandlance, polychaete, sculpin, hake, butterfish, and krill) and the MDS axes where vector length and direction reflects taxon frequency. See caption on Figure 5 for more explanations on MDS plots and vector overlays.

Chick prey items were identified visually in 12 different field observation prey categories: butterfish, euphausiid, hake, hake or herring, herring, pollock, polychaete, sandlance, sculpin, squid, unidentified fish, and unidentified invertebrate (see Table S2 for descriptions). The prey categories ‘unidentified fish’ and ‘unidentified’ which were observed in 27.8% and 0.2% of deliveries were omitted from analysis, as there was no comparable category in our DNA data. We also did not include ‘hake or herring’ data, as this is an ambiguous category contributing to only 10 deliveries (1.75%). ‘pollock’ (0.2%) and ‘squid’ (0.5%) were not identified in DNA-derived diet so they were also excluded. This resulted in 433 independent deliveries of prey made by adults to chicks. Of 65 chick fecal samples, 54 contained taxa that could be assigned to one of the seven available feeding observation prey categories (10 DNA-identified taxa, Figure 6; Table S2). The two methods of assessment resulted in significant differences in the composition of chick diet (ANOSIM, Global R 0.398, p<0.001, Figure 7). Our molecular analysis did not identify squid or pollock in chick diet, although both were seen in 2009 field observations. However, we found DNA from two fish species (Bluefish, *Pomatomus saltatrix* and Rock gunnel, *Pholis gunnellus*), that had been identified in chick diet in previous years of field observations on Machias Seal Island but not in 2009, as well as fish species never before identified through field observations (Giant wrymouth (*Cryptacanthodes maculatus*), Threadfin rockling (*Gaidropsarus ensis*), Atlantic cod (*Gadus, morhua*), Scallop snailfish (*Liparis inquilinus*), Haddock (*Melanogrammus aeglefinus*), and Acadian redfish (*S. fasciatus*) (Table S2)). 

## Discussion

Our use of multi-locus pyrosequencing to reconstruct the diets of puffins and one of their primary food sources allowed for a more complete puffin food chain than known to date (Figure 3). The number and diversity of taxa identified from our molecular evaluation of diet is far greater than, though still consistent with, conventional diet studies in both herring (Table S3) and puffins (Figure 6; Table S2). Many invertebrate taxa with planktonic life stages detected in herring diet were also found in puffin diet. We also found chick and adult puffins to have similar diets, although adult samples tended to have a higher proportion of invertebrate taxa.. This research demonstrates the general utility of next generation sequencing with multiple markers and highlights the use of this powerful tool for food web reconstruction.

### Comparison of Diet Methods

The diet of chicks when assessed by molecular methods is more highly resolved taxonomically than when assessed by conventional field observations (Figure 6, Table S2). Gulf of Maine puffin chick diet is known from years of observing adults carrying prey crosswise in the bill and delivering them to chicks in burrows [23]. Prey types are often reported in broad categories, such as ‘polychaete’ or ‘sculpin’. Through fecal analysis we can attribute ‘polychaetes’ to include the Ragworm and the Slender ragworm, ‘sculpin’ includes the Sea raven (*Hemitripterus americanus*) and Grubby (*Myoxocephalus aenaeus*), and add *Thysanoessa* sp. to the ‘krill’ category, which was previously thought to comprise only Northern krill. 

We saw a similar improvement on the taxonomic resolution of herring prey using a DNA-based approach compared to stomach content analyses. In published studies of juvenile herring diet (Table S3), prey items are also classified in broad categories such as: cladocerans; eggs from fish, crustaceans, or decapod crustaceans; barnacle or decapod larvae; a class of tunicate; a protozoan family; and krill, with the exception of one krill species (*Thysanoessa raschii*) and 12 copepod species and genera. Through DNA sequencing of stomach contents we identified three cladoceran species, eight fish species/genera, three crab species/genera, one shrimp species, and a species and genus each of both barnacle and krill.

Our molecular method identified prey not detected or not accurately identified by conventional methods. DNA from insects, echinoderms, amphipods, bivalves, gastropods, polychaetes, and cnidarians was present in herring stomach contents. None of these groups had been observed in previous studies, which may either be due in part to a change in herring diet in the 30 years since the latest published study was conducted, or reflect the increased resolution of a DNA-derived diet. Some taxa, however, would have never been detected in herring diet, such as the Common jellyfish (*Aurelia aurita*), as this prey would be digested completely, leaving nothing for identification. We also detected several taxa that were either visually misidentified as common prey or absent from the diet in 15 years of field observations of puffins (Giant wrymouth, Threadfin rockling, Atlantic cod, Hippolytidae (shrimp), Scallop snailfish, Haddock, and Acadian redfish). As many of the fish caught by puffins are small (~5-15cm), accurate identification can be difficult in the field. Shrimp from the Hippolytidae family, for instance can be easily mistaken for krill, a common prey item. While shrimp were not recorded in chick diet during 2009 field observations, one species (*Pandalus montagui*, Pandalidae) was found as a dropped prey item in the colony that year (personal observation), offering further evidence that field observations suffer from misidentification error. 

Failure to detect or misidentification of prey in predator diet can be a substantial hindrance to our understanding of how components of an ecosystem interact. For example, identifying commercially fished species consumed by puffins (Cod, Haddock, and Redfish) is important for the effective management of these stocks, as the impact of non-human predators on fish has historically been severely underestimated [3]. The natural mortality rate of herring used in stock assessment models, for example, was less than 25% of the estimated consumption by mammals, piscivorous fish, and seabirds [3]. Explicit consideration of the links between exploited species and the rest of the ecosystem, termed ecosystem-based management, has superseded historical stock-based resource management [1,2,4,5,6,7]. As predators, puffins can play a part in the population dynamics of their prey and therefore diet studies that do not suffer from misidentification of prey are preferred and their role needs to be incorporated into models of ecosystem function. 

### Effect of Secondary Consumption

While field observations did not accurately identify or quantify chick diet, they did provide a necessary framework from which to interpret our molecular diet data. The fish and shrimp species found in chick diet but not observed in the field were most likely misidentified as one of the more common fish prey or as krill, in the case of shrimp. However, the DNA-identified taxa without visual equivalents that had not been identified through field observations are either a result of secondary consumption, or are newly-identified puffin prey. Adult puffins catch and hold prey in their bills as they propel themselves underwater and upon return to the colony they drop the intact prey in their nest burrow, making very unlikely any transfer of non-targeted prey to their chicks. The copepod *A. longiremis* as well as larval stages of the Crevice brittlestar (*Ophiopholis aculeata*) and Paguridae crabs are components of plankton that are too small to be target prey for provisioning puffins. The adult forms of these species have never been seen in chick diet, nor do they look sufficiently similar to taxa that have been identified in the field that they could have been be mistaken (“visual equivalents”). Further, these three taxa were also detected in herring samples, implicating secondary consumption as the explanation for these records. 

With evidence for secondary consumption in the prey shared between chicks and herring only, we can consider removing barnacles (*Balanus* sp., *Semibalanus balanoides*), copepods (Calanoida, *Temora longicornis*, *Acartia longiremis*), cladocerans (*Evadne nordmanni*, E. *spinifera*), crabs (Great spider crab, Paguridae), and a brittlestar (Crevice brittlestar) from our picture of the diet of chicks (Figure 2). The remaining prey types of chicks (fish, krill, polychaetes, and shrimp) are known prey or have visual equivalents and can therefore be proposed as the true shared resources between herring and puffins (Figure 3). If the prey items found in chick feces were considered in isolation from those in herring diet, we would have made erroneous conclusions about foraging biology and diet of puffin chicks. The inclusion of herring diet in our study was critical to understanding puffin chick diet due to the confounding effect of secondary consumption.

### Comparison of Chick and Adult Diet

Fecal DNA analysis has shown that the community of taxa in adult puffin samples is indistinguishable from that of chicks (Figure 4, Figure 5), supporting results of a 2006 stable isotope analysis study of the same puffin population [24]. Central-place foraging theory leads to the expectation that adult and chick diets should differ, a phenomenon observed in other seabirds [48,49,50,51,52]. Though supported by stable isotope data, we question whether our data lacked the power to test this idea conclusively. Future studies should account for the difficulty in amplifying fecal DNA and collect many samples in appropriate time periods.

Adult puffins forage at sea and do not leave identifiable components of prey in feces or in the form of a pellet. While stomach content analyses of dead or sacrificed birds have been conducted in European populations, no study has yet identified the prey of Gulf of Maine adult puffins [53]. As a result, chick diet has been used as a best estimate of adult diet. Our results provide the first ever assessment of puffin adults in the Gulf of Maine and, in addition to stable isotope data, support the use of chick diet as an acceptable proxy for adult diet.

### Foraging Habitat

 This DNA-based method identified many herring prey to species or genus level, allowing us to draw conclusions about herring foraging habitat and behaviour based on the biology and selection of prey on which they feed, which in turn can be used to understand how changes at the base of the food chain may affect puffins.

Juvenile herring are considered as “plankton feeders” (Table S3 and references therein) and components of the pelagic food web. In the summer months, schools of juvenile herring occupy discrete small home ranges in and around inlets and bays on the Gulf of Maine coast [29]. Our data support previous observations of juvenile herring occupying near-shore habitats as we identified the coastal and ‘shallow water’ copepods *Acartia longiremis* [54] and *T. longicornis* [55], and the fresh- or brackish water cladoceran *Pleopsis polyphemoides* [56] in our herring diet samples. We also identified pelagic prey, as both species of krill (Northern krill and *Thysanoessa inermis*) detected in herring diet have been shown to be absent from areas where bottom depth was less than 45 metres [57]. However, our data show herring to consume non-planktonic prey from non-pelagic habitats. For instance, *Corophium volutator* (Figure 2), is a mud flat-living amphipod [58], *Lacuna* sp. has one regional epifaunal representative [47], and the Ragworm is a shallow-sediment dweller [46]. 

Juvenile herring are believed to capture prey items actively as opposed to passively ingesting whatever floats into their open mouth [29]. It has been shown that year-class size is unrelated to primary production [59] and the amount of food in herring stomachs is not correlated with the abundance of zooplankton in the water [30]. The composition of herring diet is therefore a matter of choice and not a random sample of species available in the environment. Further, juvenile herring remain in the same general locality during the principal growing period (June to August) and the body condition of these distinct schools of herring is consistent across years [29]. We offer two potential explanations for how we might observe herring prey from a range of habitats (near shore and benthic to planktonic and pelagic) while herring themselves occupy small and discrete home ranges. The full diversity of prey taxa might be available at each location if bottom topography is sufficiently variable within such a short distance to support food chains based on both benthic and pelagic production. Alternatively, if each location used by juvenile herring provides a different range of prey, foraging puffins must be exploiting different patches of juvenile herring to produce the varied prey community we have detected in their stomach contents. These alternative interpretations provide competing predictions about both spatial ecology of juvenile herring and foraging behaviour of breeding puffins that should be amenable to testing in the field.

Taxa identified in adult puffin diet may also provide evidence for foraging habitat. Several taxa present in adult diet occupy freshwater (a rotifer (Bdelloidea), a diatom (*Pinnularia* sp.), an oligochaete (*Nais elinguis*)) and intertidal (an amphipod (*Gammarellus angulosus*) and Rockweed (*Ascophyllum nodosum*)) habitats. The presence of these taxa may reflect a coastal foraging habitat of adult puffins, or secondary consumption that was not confirmed due to insufficient overlap between adult puffin and herring diet samples. We recommend future DNA-based diet studies aim to control for confounding secondary consumption through the investigation of prey diet but also by ensuring sufficient number and overlap of predator diet samples across the sampling period so that temporal differences in prey availability are not confused with secondary consumption.

### Methods

We wished to encompass as much taxonomic diversity as possible in this food chain study so we chose two loci with published degenerate primers which produce small amplicons. The combination of universal primers (chosen in regions of non-variable DNA) and short amplicons (necessary to function effectively on degraded DNA) did pose some problems resolving a few taxa. For both 16S and CO1, the fragment produced from the universal primers was not sufficiently polymorphic to identify which of the two Gulf of Maine *Ammodytes* species (*dubius* or *americanus*) were present in our diet samples. Additionally, there were many instances where prey could not be resolved to species or genus level because those species had not been previously sequenced at our loci of interest. In several cases the taxon assignment was so general that it was not useful in describing diet at all (e.g.: the first entries in Table S1) or no assignment could be made, which may be a due to either considerable deficiencies in the reference database (GenBank) or sequencing error. There were stark differences in the type, number, and coverage of taxa that each marker identified. Marker choice has a considerable impact on how diet is assessed as only 20% of all animal taxa were detected by both markers. Dietary data, and the interpretation of how an organism relates to the greater food web, are skewed by the effectiveness of a marker to amplify various groups of organisms. The variability in prey taxa coverage between loci (Figure 1) is a methodological bias that strongly supports a multi-locus approach.

**Figure 8:**
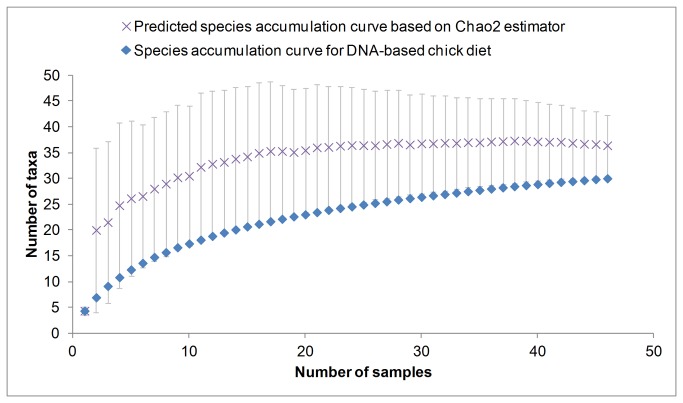
Species accumulation curve for DNA-based chick diet. Only samples with >50 sequences from kingdom Animalia were included. Crosses with standard deviation error bars represent Chao2 S extrapolator for prediction of the true total number of taxa present in samples.

 Although our method identified many more taxa than field observations, an estimated 6 species are present but undetected in our samples (PRIMER Choa2 S extrapolator, Figure 8). Our data provide overwhelming support for the use of multiple barcoding markers, particularly when no *a priori* knowledge of diet is available, to increase the likelihood of capturing the true assemblage of prey present in predator diet and improve the chance of accurately describing the trophic interactions within a food web. 

There was sufficient overlap in chick and herring samples to confidently identify secondary consumption in puffin chicks, however this is not true for adult puffin diet. We were unable to detect the full complement of prey for all components of this food chain (Figure 8). It is also important to note that puffins feed on fish species other than herring, which may contribute to the incidence of secondary consumption. However, we predict the effect of secondary consumption from non-herring prey is limited as both field observations and molecular data (Figure 6) show herring as the predominant food of puffins in this sampling time period.

## Conclusions

This study represents the first simultaneous molecular investigation into the diet of multiple components of a food chain. Both puffin and herring diet were described with more diversity at a higher taxonomic resolution with our molecular approach compared to conventional methods, enhancing our knowledge of the biology of and interactions between these animals. The sensitivity of these techniques to detect the prey of prey is an important consideration for molecular scatology, particularly when *de novo* diet assembly with universal primers is concerned. We suggest that results of DNA-based diet studies be viewed from the perspective of a food chain, rather than simply diet, due to the effect of secondary consumption. Further, because of the considerable discrepancies in the types, coverage, and frequency of occurrence of prey taxa between markers, we recommend the use of multiple barcoding markers for taxon identification.

 The broad significance of our study is that both herring and puffins, previously considered to be part of the planktonic food web only, prove instead to be part also of the inshore or intertidal food web, in which benthic production also plays a role. Secondary consumers in this web can no longer be assumed to derive their energy and nutrients from planktonic production alone. Contaminant studies will need to take into account a possibly greater role for benthic and intertidal pathways leading to higher trophic levels of the Gulf of Maine food web. 

What a predator eats is perhaps the most ecologically important information we can learn about an animal. These predator-prey relationships are used to describe food chains, which provide the framework from which we understand how energy flows through an ecosystem. To improve our capacity to effectively manage and conserve marine ecosystems, we propose a multi-trophic, multi-locus, pyrosequencing approach for a comprehensive description of a food web from which we can interpret ecosystem functioning.

## Supporting Information

Table S1 **All identified taxa from DNA sequencing herring stomach contents and chick and adult puffin fecal samples collected within the common sampling period (13 June to 29 July).** Frequency of occurrence (FOO) is listed for taxa found in samples with at least 50 sequences per marker per sample. Taxa identified in samples that produced less than 50 sequences receive an NF (no FOO). When a taxon was identified (as a MOTU) with both markers, the total number of samples was used for FOO calculation (adult n=39, chick n=46, herring n=37) otherwise sample size of FOO calculation depends on the marker from which a taxon was identified.
(DOCX)

Table S2 **Description of prey categories used in field observations for 2009 MSI puffin chick diet and corresponding DNA taxa.** Number of prey items identified from field observations of provisioning adults on Machias Seal Island 1995-2010 (LEFT, [23]). Description of prey categories identified in 2009 field observations and the associated DNA-identified taxa (RIGHT). Bolded prey: observed with one method only.
(DOCX)

Table S3 **Summary of juvenile herring diet from published stomach content analyses.** Most abundant or frequently observed prey types (numbers) or copepods species/genera (letters) are listed in ascending order.
(DOCX)

Table S4 **Summary of study sample sizes.**
(DOCX)
