# Supplementary material for: From Puffins to Plankton: A DNA-Based Analysis of a Seabird Food Chain in the Northern Gulf of Maine
Source: PLoS One. 2013 Dec 16;8(12):e83152. doi: 10.1371/journal.pone.0083152 (PMC3865145; doi:10.1371/journal.pone.0083152)
Supplement: Table S1 — All identified taxa from DNA sequencing herring stomach contents and chick and adult puffin fecal samples collected within the common sampling period (13 June to 29 July). Frequency of occurrence (FOO) is listed for taxa found in samples with at least 50 sequences per marker per sample. Taxa identified in samples that produced less than 50 sequences receive an NF (no FOO). When a taxon was identified (as a MOTU) with both markers, the total number of samples was used for FOO calculation (adult n=39, chick n=46, herring n=37) otherwise sample size of FOO calculation depends on the marker from which a taxon was identified. (DOCX) [file pone.0083152.s001.docx]

**Table S1: All identified taxa from DNA sequencing herring stomach contents and chick and adult puffin fecal samples collected within the common sampling period (13 June to 29 July).**

|  |  | **Frequency of Occurrence (%)** | | | | | |
| --- | --- | --- | --- | --- | --- | --- | --- |
|  |  | **Adult** | | **Chick** | | **Herring** | |
|  |  | n=39 | | n=46 | | n=37 | |
|  |  | **16S** | **CO1** | **16S** | **CO1** | **16S** | **CO1** |
| **Taxon and Marker** | **Taxonomic Hierarchy** | n=29 | n=18 | n=41 | n=13 | n=25 | n=23 |
| Opisthokonta, CO1 |  | not present | | 7.7 | | not present | |
| Coelomata, CO1 |  | 27.8 | | 7.7 | | 52.2 | |
| Eukaryota, CO1 |  | 27.8 | | 7.7 | | 21.7 | |
| Eumetazoa, CO1 |  | 16.7 | | not present | | 30.4 | |
| *Nais elinguis*, 16S | Annelida, Clitellata | 17.6 | | not present | | not present | |
| Naididae, 16S | Annelida, Clitellata | NF | | not present | | not present | |
| Polychaeta, CO1 | Annelida, | not present | | not present | | 4.3 | |
| Capitellidae, CO1 | Annelida, | 5.6 | | not present | | 8.7 | |
| *Nephtys* sp., CO1 | Annelida, Polychaeta | not present | | not present | | 4.3 | |
| *Hediste diversicolor*, Ragworm, 16S | Annelida, Polychaeta | 70.6 | | 51.2 | | 12 | |
| *Nereis pelagica*, Slender ragworm, 16S | Annelida, Polychaeta | 11.8 | | 34.1 | | not present | |
| Phyllodocidae, CO1 | Annelida, Polychaeta | not present | | not present | | 13 | |
| Pancrustacea, crustaceans+hexapods, both | Arthropoda, | 3.7 | | not present | | 5.4 | |
| *Evadne nordmanni*, cladoceran, both | Arthropoda, Branchiopoda | 14.8 | | 15.2 | | 43.2 | |
| *Evadne spinifera*, cladoceran, CO1 | Arthropoda, Branchiopoda | 94.4 | | 61.5 | | 100 | |
| *Pleopsis polyphemoides*, cladoceran, 16S | Arthropoda, Branchiopoda | not present | | not present | | 8 | |
| Neoptera, winged insect, 16S | Arthropoda, Insecta | 11.8 | | 7.3 | | 56 | |
| Brachycera, fly, 16S | Arthropoda, Insecta | not present | | 7.3 | | not present | |
| *Liposcelis decolor*, Book louse, 16S | Arthropoda, Insecta | NF | | not present | | not present | |
| *Corophium volutator*, mud shrimp, CO1 | Arthropoda, Malacostraca | not present | | not present | | 4.3 | |
| *Gammarellus angulosus*, amphipod, 16S | Arthropoda, Malacostraca | NF | | not present | | not present | |
| Alpheoidea, shrimp, CO1 | Arthropoda, Malacostraca | not present | | not present | | 4.3 | |
| Caridea, shrimp, both | Arthropoda, Malacostraca | not present | | not present | | 12 4.3, (13.5) | |
| *Eualus fabricii*, Arctic eualid, both | Arthropoda, Malacostraca | not present | | not present | | 8 4.3, (5.4) | |
| Hippolytidae, shrimp, 16S | Arthropoda, Malacostraca | 52.9 | | 22 | | 92 | |
| Pandalidae, shrimp, 16S | Arthropoda, Malacostraca | not present | | not present | | 4 | |
| *Meganyctiphanes norvegica*, Northern krill, 16S | Arthropoda, Malacostraca | 35.3 | | 2.4 | | 20 | |
| *Thysanoessa* sp., krill, 16S | Arthropoda, Malacostraca | 5.9 | | 2.4 | | 16 | |
| Majoidea, crab, 16S | Arthropoda, Malacostraca | not present | | not present | | 4 | |
| *Cancer* sp., crab, 16S | Arthropoda, Malacostraca | 5.9 | | not present | | not present | |
| *Homarus* *americanus*, Lobster, 16S | Arthropoda, Malacostraca | NF | | not present | | not present | |
| *Hyas araneus*, Great spider crab, 16S | Arthropoda, Malacostraca | 29.4 | | 36.6 | | 84 | |
| Paguridae, hermit crab, 16S | Arthropoda, Malacostraca | not present | | 2.4 | | 24 | |
| *Pagurus* sp., hermit crab, 16S | Arthropoda, Malacostraca | not present | | not present | | 4 | |
| *Carcinus maenas*, Green crab, 16S | Arthropoda, Malacostraca | not present | | not present | | 4 | |
| Calanoida, copepod, both | Arthropoda, Maxillopoda | NF | | 4.3 | | 2.7 | |
| *Acartia longiremis*, copepod, 16S | Arthropoda, Maxillopoda | not present | | 2.4 | | 12 | |
| *Calanus finmarchicus*, copepod, 16S | Arthropoda, Maxillopoda | NF | | not present | | 4 | |
| *Calanus glacialis*, copepod, 16S | Arthropoda, Maxillopoda | 5.9 | | not present | | not present | |
| *Calanus hyperboreus*, copepod, CO1 | Arthropoda, Maxillopoda | not present | | not present | | 4.3 | |
| Euchaetidae, copepod, CO1 | Arthropoda, Maxillopoda | not present | | not present | | 8.7 | |
| *Metridia longa*, copepod, 16S | Arthropoda, Maxillopoda | 5.9 | | not present | | 8 | |
| *Metridia lucens*, copepod, 16S | Arthropoda, Maxillopoda | not present | | not present | | 28 | |
| *Temora longicornis*, copepod, CO1 | Arthropoda, Maxillopoda | 66.7 | | 84.6 | | 87 | |
| *Semibalanus balanoides*, barnacle, both | Arthropoda, Maxillopoda | 3.7 | | 4.3 | | 13.5 | |
| *Balanus* sp., barnacle, both | Arthropoda, Maxillopoda | 11.1 | | 8.7 | | 45.9 | |
| *Caligus elongatus*, parasitic copepod, 16S | Arthropoda, Maxillopoda | not present | | not present | | 4 | |
| Elopocephala, fish, CO1 | Chordata, Actinopterygii | 5.6 | | not present | | not present | |
| Clupea harengus, Atlantic herring, both | Chordata, Actinopterygii | 100 | | 100 | | 91.9 | |
| *Enchelyopus cimbrius*, Fourbeard rockling, 16S | Chordata, Actinopterygii | not present | | not present | | 4 | |
| *Gadus morhua*, Atlantic cod, 16S | Chordata, Actinopterygii | NF | | 2.4 | | not present | |
| *Gaidropsarus ensis*, Threadfin rockling, 16S | Chordata, Actinopterygii | not present | | 4.9 | | not present | |
| *Melanogrammus aeglefinus*, Haddock, 16S | Chordata, Actinopterygii | 17.6 | | 14.6 | | 4 | |
| *Urophycis tenuis*, White hake, both | Chordata, Actinopterygii | 14.8 | | 15.2 | | 10.8 | |
| *Gasterosteus wheatlandi*, Blackspotted stickleback, CO1 | Chordata, Actinopterygii | not present | | not present | | 4.3 | |
| Perciformes 1, fish, 16S | Chordata, Actinopterygii | NF | | not present | | not present | |
| Perciformes 2, fish, 16S | Chordata, Actinopterygii | 5.9 | | not present | | not present | |
| *Ammodytes* sp., sandlance, both | Chordata, Actinopterygii | 33.3 | | 37 | | 5.4 | |
| *Cryptacanthodes giganteus*, Giant wrymouth, 16S | Chordata, Actinopterygii | not present | | 2.4 | | not present | |
| *Pholis gunnellus*, Rock gunnel, 16S | Chordata, Actinopterygii | 11.8 | | 7.3 | | not present | |
| *Pomatomus saltatri*, Bluefish, 16S | Chordata, Actinopterygii | not present | | 2.4 | | not present | |
| *Peprilus triacanthus*, Butterfish, 16S | Chordata, Actinopterygii | NF | | 17.1 | | not present | |
| *Myoxocephalus aenaeus*, Grubby, CO1 | Chordata, Actinopterygii | 5.6 | | 7.7 | | 13 | |
| *Hemitripterus americanus*, Sea raven, 16S | Chordata, Actinopterygii | 5.9 | | 9.8 | | not present | |
| *Liparis inquilinus*, Scallop snailfish, 16S | Chordata, Actinopterygii | 5.9 | | 4.9 | | NF | |
| *Sebastes fasciatus*, Acadian redfish, both | Chordata, Actinopterygii | 33.3 | | 4.3 | | 13.5 | |
| *Obelia longissima*, Hydrozoan, CO1 | Cnidaria, Hydrozoa | not present | | not present | | 8.7 | |
| *Aurelia aurita*, Common jellyfish, CO1 | Cnidaria, Scyphozoa | not present | | not present | | 4.3 | |
| *Asterias rubens*, Common sea star, CO1 | Echinodermata, Asteroidea | 5.6 | | not present | | 4.3 | |
| Ophiuroidea, brittlestar, 16S | Echinodermata, Ophiuroidea | not present | | not present | | 8 | |
| *Ophiopholis aculeata*, Crevice brittlestar, 16S | Echinodermata, Ophiuroidea | not present | | 2.4 | | 8 | |
| *Mytilus* sp., mussel, 16S | Mollusca, Bivalvia | NF | | not present | | 8 | |
| *Ensis directus*, Atlantic jacknife clam, CO1 | Mollusca, Bivalvia | not present | | not present | | 21.7 | |
| *Lacuna* sp., chink shell, 16S | Mollusca, Gastropoda | not present | | not present | | 16 | |
| Cephalothricidae, ribbon worm , 16S | Nemertea, Anopla | not present | | not present | | NF | |
| Rotifera, 16S | Rotifera, | not present | | not present | | 4 | |
| Bdelloidea, CO1 | Rotifera, Eurotatoria | 5.6 | | not present | | not present | |
| *Synchaeta* sp., rotifer, CO1 | Rotifera, Eurotatoria | not present | | not present | | 13 | |
| Alteromonadales, CO1 | Bacteria, Proteobacteria | not present | | 7.7 | | not present | |
| Bacilli, 16S | Bacteria, Firmicutes | 29.4 | | 26.8 | | not present | |
| *Mycoplasma* *synoviae*, 16S | Bacteria, Firmicutes | 23.5 | | 24.4 | | 8 | |
| *Mycoplasma* sp., 16S | Bacteria, Firmicutes | 17.6 | | 31.7 | | 8 | |
| Psychrobacter arcticus, CO1 | Bacteria, Proteobacteria | 5.6 | | not present | | not present | |
| Oomycetes, CO1 | Chromalveolata Heterokontophyta | 11.1 | | 7.7 | | 4.3 | |
| Bacillariophyta, diatom, CO1 | Chromalveolata Heterokontophyta | not present | | not present | | 4.3 | |
| *Pinnularia isselana*, diatom, CO1 | Chromalveolata Heterokontophyta | 5.6 | | not present | | not present | |
| *Nitzschia palea*, diatom, CO1 | Chromalveolata Heterokontophyta | 5.6 | | not present | | not present | |
| *Ascophyllum nodosum*, Rockweed, CO1 | Chromalveolata Heterokontophyta | 5.6 | | not present | | not present | |
| *Pythium* sp., water mould, CO1 | Chromalveolata Heterokontophyta | not present | | 7.7 | | not present | |
| *Pythium tracheiphilum*, water mould, CO1 | Chromalveolata Heterokontophyta | 5.6 | | not present | | not present | |
| *Clostridium perfringens*, 16S | Bacteria, Schizomycetes | NF | | not present | | not present | |
| *Coccomyxa* sp., green alga, CO1 | Plantae, Chlorophyceae | 11.1 | | 15.4 | | not present | |
| *Nebela* sp., CO1 | Protozoa, Lobosa | 11.1 | | 15.4 | | not present | |

Frequency of occurrence (FOO) is listed for taxa found in samples with at least 50 sequences per marker per sample. Taxa identified in samples that produced less than 50 sequences receive an NF (no FOO). When a taxon was identified (as a MOTU) with both markers, the total number of samples was used for FOO calculation (adult n=39, chick n=46, herring n=37) otherwise sample size of FOO calculation depends on the marker from which a taxon was identified.
