# Supplementary material for: From Puffins to Plankton: A DNA-Based Analysis of a Seabird Food Chain in the Northern Gulf of Maine
Source: PLoS One. 2013 Dec 16;8(12):e83152. doi: 10.1371/journal.pone.0083152 (PMC3865145; doi:10.1371/journal.pone.0083152)
Supplement: Table S2 — Description of prey categories used in field observations for 2009 MSI puffin chick diet and corresponding DNA taxa. Number of prey items identified from field observations of provisioning adults on Machias Seal Island 1995-2010 (LEFT, [23]). Description of prey categories identified in 2009 field observations and the associated DNA-identified taxa (RIGHT). Bolded prey: observed with one method only. (DOCX) [file pone.0083152.s002.docx]

**Table S2: Description of prey categories used in field observations for 2009 MSI puffin chick diet and corresponding DNA taxa.**

|  |  | **2009 Diet Method Comparison** | |
| --- | --- | --- | --- |
| **Field Observation Prey Category** | **Observed 1995-2010 (# Items)** | **Field-Observed Prey Category Description** | **DNA-Identified Taxa** |
| Butterfish | 350 | *Peprilus triacanthus* | *Peprilus triacanthus* |
| Krill | 5121 | Any krill (Euphausiacea) but typically *Meganyctiphanes norvegica* | *Meganyctiphanes norvegica*, *Thysanoessa* sp. |
| Hake | 4580 + 270 (larval) | *Urophycis* *tenuis*, *U*. *chuss*, and *Enchelyopus* *cimbrius* | *Urophycis* *tenuis*, *Urophycis* sp., and *Enchelyopus* *cimbrius* |
| Hake or Herring | 1304 | Fish belonging to ‘Hake’ group or *Clupea* *harengus* | N/A |
| Herring | 6328 + 126 (larval) | *Clupea* *harengus* | *Clupea* *harengus* |
| Pollock | 63 | ***Pollachius* *virens*** | Not detected |
| Polychaete | 132 | Any Polychaete (Polychaeta) | *Nereis* *pelagica*, *Hediste* *diversicolor*, *Polychaeta* |
| Sandlance | 2759 + 222 (larval) | Typically *Ammodytes* *americanus*, potentially *A.* *dubius* | *Ammodytes* sp |
| Sculpin | 5 | Fish that look like sculpin (Cottoidea) | *Myoxocephalus* *aenaeus*, *Hemitripterus* *americanus* |
| Squid | 85 | **Any squid** | Not detected |
| Unidentified Fish | 3400 + 2480 (larval) | Any unknown fish | N/A |
| Unidentified | 723 | Any unknown item | N/A |
| Rock Eel/Gunnel | 1 | Not detected | ***Pholis* *gunnellus*** |
| Bluefish | 2 | Not detected | ***Pomatomus* *saltatrix*** |
| Crustacean | 3 | Not detected | Multiple species |
| Lumpfish | 3 | Not detected | |
| Stickleback | 3 | Not detected | |
| Atlantic Saury | 4 | Not detected | |
| Fish Scrap | 17 | Not detected | N/A |
| Eelpout | 36 | Not detected | |
| Earthworm | 42 | Not detected | |
| N/A | N/A | Not detected | **Hippolytidae** |
| N/A | N/A | Not detected | ***Melanogrammus aeglefinus*** |
| N/A | N/A | Not detected | ***Liparis inquilinus*** |
| N/A | N/A | Not detected | ***Gaidropsarus ensis*** |
| N/A | N/A | Not detected | ***Sebastes fasciatus*** |
| N/A | N/A | Not detected | ***Cryptacanthodes giganteus*** |
| N/A | N/A | Not detected | ***Gadus morhua*** |
| N/A | N/A | Not detected | **Brachycera** |
| N/A | N/A | Not detected | **Neoptera** |

Number of prey items identified from field observations of provisioning adults on Machias Seal Island 1995-2010 (LEFT, [23]). Description of prey categories identified in 2009 field observations and the associated DNA-identified taxa (RIGHT). Bolded prey: observed with one method only.
