# Supplementary material for: From Puffins to Plankton: A DNA-Based Analysis of a Seabird Food Chain in the Northern Gulf of Maine
Source: PLoS One. 2013 Dec 16;8(12):e83152. doi: 10.1371/journal.pone.0083152 (PMC3865145; doi:10.1371/journal.pone.0083152)
Supplement: Table S3 — Summary of juvenile herring diet from published stomach content analyses. Most abundant or frequently observed prey types (numbers) or copepods species/genera (letters) are listed in ascending order. (DOCX) [file pone.0083152.s003.docx]

**Table S3:** **Summary of juvenile herring diet from published stomach content analyses.**

| **Reference** | | [29] | [30] | [26] | [27] | | [28] |
| --- | --- | --- | --- | --- | --- | --- | --- |
| **Herring length** | | 90-110mm | 60-103mm | >50mm | 97-207mm | 110-212mm | 110-150mm |
| **Year and Season** | | 1933 summer | 1958 spring-fall | 1968 spring | 1968 spring | 1968 summer | 1977-1980 year round |
| **Identified prey** | |  |  |  |  |  |  |
| Crustaceans |  |  |  |  |  |  |  |
|  | Euphausiacea (krill) |  |  |  |  | 8 |  |
|  | *Thysanoessa raschii* |  |  |  |  |  | 1 |
|  | Cladocera (water fleas) |  |  |  |  | 3 |  |
|  | Copepoda (copepods)* | 1 | 1 | 1 | 2 | 1 |  |
| Eggs |  |  | 2 |  |  |  |  |
|  | Fish | 2 |  | 4 |  |  |  |
|  | Crustacea |  | 3 |  |  |  |  |
|  | Decapoda |  |  |  | 4 |  |  |
| Crustacean larvae |  |  |  |  |  | 5 |  |
|  | Cirripedia (barnacles) |  |  | 2 | 1 | 6 |  |
|  | Decapoda |  |  |  | 5 | 7 |  |
|  | Copepodite |  |  | 5 |  |  |  |
| Protozoans |  |  |  |  |  |  |  |
|  | Tintinnidae |  |  | 4 |  |  |  |
| Tunicates |  |  |  |  |  |  |  |
|  | Appendicularia |  |  |  | 3 | 2 |  |
| *Copepoda |  |  |  |  |  |  |  |
|  | *Calanus finmarchicus* |  |  |  | A | A |  |
|  | *Calanus* spp. | B |  |  |  |  |  |
|  | *Temora longicornis Temora* spp. | A |  |  |  | C |  |
|  | *Psuedocalanus minutus* |  |  | A | B | B |  |
|  | Harpacticoida/Cyclopoida |  |  | B |  |  |  |
|  | *Oithona* sp. |  |  | C |  |  |  |
|  | *Acartia* sp. | C | B | D | C | E |  |
|  | *Tortanus discauatus* |  |  |  |  | D |  |
|  | *Centropages typicus* |  |  |  |  | F |  |
|  | *Centropages hamatus* |  |  |  |  | G |  |
|  | *Eurytemora* sp. |  | A |  |  |  |  |

Most abundant or frequently observed prey types (numbers) or copepods species/genera (letters) are listed in ascending order.
