# Supplementary material for: From Puffins to Plankton: A DNA-Based Analysis of a Seabird Food Chain in the Northern Gulf of Maine
Source: PLoS One. 2013 Dec 16;8(12):e83152. doi: 10.1371/journal.pone.0083152 (PMC3865145; doi:10.1371/journal.pone.0083152)
Supplement: Table S4 — Summary of study sample sizes. (DOCX) [file pone.0083152.s004.docx]

**Table S4: Summary of study sample sizes.**

|  | **Collected** | **Amplifications** | **# Successful** | **Unique Samples** | **Common Sample Period + >50 Sequences/Sample** |
| --- | --- | --- | --- | --- | --- |
| **Adult** | 146 | 86 |  | 64^a,e^ | 39^b^ |
| 16S |  | 60 | 55 |  | 29^c^ |
| CO1 |  | 26 | 24 |  | 18 |
|  |  |  |  |  |  |
| **Chick** | 91 | 89 |  | 65^a,d,e^ | 46^b^ |
| 16S |  | 63 | 62 |  | 41^c^ |
| CO1 |  | 26 | 25 |  | 13 |
|  |  |  |  |  |  |
| **Herring** | 77 | 73 |  | 44^a,e^ | 37^b^ |
| 16S |  | 41 | 40 |  | 25 |
| CO1 |  | 32 | 32 |  | 23 |
| 16SNB |  | 18 | 18 |  |  |
| Figure 1^a^  Figure 2^b^  Figure 3+4^c^ (only 17 diet-informative 16S adult)  Figure 5+6^d^ (11 omitted due to lack of shared taxa)  Figure 7^e^  NB=No blocking primer | | | | | |
